# Introgression of *opaque2* into Waxy Maize Causes Extensive Biochemical and Proteomic Changes in Endosperm

**DOI:** 10.1371/journal.pone.0158971

**Published:** 2016-07-08

**Authors:** Zhiqiang Zhou, Liya Song, Xiaoxing Zhang, Xinhai Li, Na Yan, Renpei Xia, Hui Zhu, Jianfeng Weng, Zhuanfang Hao, Degui Zhang, Hongjun Yong, Mingshun Li, Shihuang Zhang

**Affiliations:** 1 Department of Crop Genetics and Breeding, Institute of Crop Science, Chinese Academy of Agricultural Sciences, Beijing, China; 2 Beijing Key Lab of Plant Resource Research and Development, Beijing Technology and Business University, Beijing, China; Huazhong University of Science and Technology, CHINA

## Abstract

Waxy maize is prevalently grown in China and other countries due to the excellent characters and economic value. However, its low content of lysine can’t meet the nutritional requirements of humans and livestock. In the present study, we introgressed the *opaque2* (*o2*) allele into waxy maize line Zhao OP-6/*O2O2* by using marker-assisted selection (MAS) technique and successfully improved the lysine content and quality of waxy maize. Transcript abundance analysis indicated that the *wx1* expression levels had no difference between Zhao OP-6/*o2o2* and Zhao OP-6/*O2O2*. However, Zhao OP-6/*o2o2* was characterized by a phenotype of hard and vitreous kernels and accumulation of protein bodies at smaller size (one third of that of parents) but in larger numbers. Biochemical analyses showed that Zhao OP-6/*o2o2* had 16.7% less free amino acids than Zhao OP-6/*O2O2*, especially those derived from glycolytic intermediates, but its content of lysine was increased by 51.6% (0.47% vs. 0.31%). The content of amylopectin was 98.5% in Zhao OP-6/*o2o2*, significantly higher than that in Zhao OP-6/*O2O2* (97.7%). Proteomic analyses indicated that *o2* introgression not only decreased the accumulation of various zein proteins except for 27-kDa γ-zein, but also affected other endosperm proteins related to amino acid biosynthesis, starch-protein balance, stress response and signal transduction. This study gives us an intriguing insight into the metabolism changes in endosperm of waxy maize introgressed with *opaque2*.

## Introduction

Waxy maize (*Zea mays* L. *sinensis* Kulesh), also known as sticky maize, is one sub-type of maize that was first discovered in Southwestern China and then prevalently grown in other Asian countries [1–3]. The endosperm of waxy maize has a high content of amylopectin (nearly 100%), and is thus characterized by high viscosity, easy digestion, and good light transmittance [4]. These excellent characters and fresh harvest make waxy maize widely used in frozen food processing, paper-making and livestock feeding industries. However, due to the limited levels and types of essential amino acids, especially lysine, the nutritional value of waxy maize is relatively low. Generally, the lysine content in maize grain should be more than 0.5% (>51 mg per gram of protein) to meet human and livestock requirements [5], but waxy maize has a lysine content of only 0.24–0.34%. By introgression of *opaque2* (*o2*) and *opaque2* modifier (*o2m*) alleles into elite maize inbred lines with marker-assisted selection (MAS) technique, the genetically modified *opaque2* maize, also known as quality protein maize (QPM), shows an improved lysine content of approximately 0.4% [6–9]. Therefore, it is of importance to breed a novel waxy maize line with high lysine content by introgressing the *o2* and *o2m* traits with MAS.

Accumulation of starch and storage proteins occurs in the developing endosperm of maize, the quality of which is contributed to by the action of the *Waxy1* (*Wx1*) and *Opaque2* (*O2*) genes [10]. The single copy 3.8-kb *Wx1* contains 14 exons and is mapped on the short arm of chromosome 9 [11,12]. Previous studies have shown that transposable elements Ac/Ds and En/Spm, deletion mutation and mutagenic ethylmethane sulfonate (EMS) mutagenesis account for the pre-mRNA splicing or translation errors and result in a low expression level of *Wx1* [13]. As a result, the granule-bound starch synthase I (GBSS I) activity of the *wx1* mutant has a decreased activity (5–95%) in amylose synthesis, leading to the low level of amylose but high level of amylopectin in maize endosperm and pollen [14,15]. O2 is a transcriptional factor that contains a basic leucine-zipper (bZIP) motif. It is specifically expressed in the developing endosperm and directly regulates the expression of 22-kDa α-zeins [16–18]. The substantial reduction of α-zeins is concomitant with increased accumulation of non-zeins, consequently accounting for the increased contents of lysine and tryptophan in maize mutants [19, 20]. In addition, a large number of studies have shown that O2 also has pleiotropic effects on the expression of non-storage proteins including ribosome-inactivating protein b-32 (RIP), cytosolic pyruvate phosphate dikinase 1 (cyPPDK1), lysine ketoglutarate reductase-saccaropine dehydrogenase (LKR-SDH), acetohydroxyacid synthase (AHAS) and Opaque2 heterodimerizing protein 1 (OHP1) [21–23]. Thus O2 as a regulator plays a crucial role in maize endosperm development by influencing the storage protein and nitrogen/carbon metabolism. Although the individual functions of *Wx1* and *O2* are well known, how these genes interact to maintain the starch-protein balance is yet unknown.

It has been reported that *o2* mutation can alter the transcriptional patterns of *Wx1* in varying degrees [24–26], but no evidence revealed the regulatory effect of *o2* on the expression of *wx1*. By backcrossing of *o2* and *o16* traits with MAS, the quality and lysine content of waxy maize have been successfully improved [27,28]. However, the molecular mechanism underlying the ameliorated amino acid composition of maize endosperm and specific kernel phenotype is yet unknown. In *o2* mutants, genes associated with glycolytic pathway, endoplasmic reticulum (ER) stress responses and amino acid synthesis demonstrated differences in transcript profiles [23], thus offering an unbiased hypothesis that some novel mechanisms play a vital role in the modification of waxy maize endosperm.

To disclose the extensive changes of endosperm metabolism when introgressed *o2* into waxy maize Zhao OP-6/*O2O2*, we constructed a set of near-isogenic lines (NILs) with QPM as the donor, and performed submicroscopic observations of the endosperm structure and biochemical analysis of the protein bodies and nutrient contents. Further proteomic analysis of immature seeds identified several specific proteins involved in metabolic pathways, such as synthesis of starch and protein, composition of amino acids and carbohydrate metabolism. Combined analysis of the results gives us an intriguing insight into the effect of integrating *o2* and *wx1* on the metabolism of maize developing endosperm.

## Materials and Methods

### Plant materials

QPM CA339 derived from pool33 (Centro Internacional de Mejoramientode Maizy Trigo, Mexico DF, Mexico) was used as the non-recurrent parent (donor). The elite waxy inbred line Zhao OP-6 (Maize Research Center of Institute of Crop Sciences, Chinese Academy of Agricultural Sciences, Beijing, China) was used as a recurrent parent (receptor). The *o2-wx* NILs, Zhao OP-6/*o2o2*, were selected from the two parents by using MAS technique. Co-dominant SSR markers phi057 and phi027 were used to select heterozygous (*O2WX/o2wx*) individuals, while the dominant SSR marker phi112 was used to detect transposable element *rbg* at BC_6_F_1_ [29]. All heterozygous genotypes were converted to the recurrent parent Zhao OP-6 through six backcrossing cycles, followed by three rounds of self-pollination. Theoretically, the *o2-wx* NILs had 99% of the recurrent parent genome, and were phenotypically uniform and genetically homogeneous after six generations of backcrossing. All of the maize materials were grown in adjacent plots in the experimental station (N40°36′ and E116°34′) of Chinese Academy of Agricultural Sciences during the summer of 2013.

A minimum of three well-filled ears of each genotype were sampled at 18 day after pollination (DAP), when the conversion of importing sucrose and amino acids into starch and storage proteins reached a high level. Ears were picked up at approximately noon, and kernels were collected from the centre of each ear. Embryos and surrounding pericarps were dissected, frozen immediately in liquid nitrogen, and stored at -80°C before use.

Mature kernels were harvested after physiological maturity and dried in a green house. To avoid biological variations, equal numbers of well-filled ears (≥ 3) were pooled and treated as one sample, and each experiment had two or more replicates.

### DNA extraction, PCR amplification, electrophoresis and genotype analysis

Seedling leaves of parents and offsprings were collected and used for DNA extraction with CTAB method. The integrity and quality of DNA were detected by electrophoresis in 1% agarose gel, and the DNA concentration was adjusted to ~100 ng/μL. Primers specific for phi057, phi027, phi112 and 100 SSR markers were adopted from the maize genome database MaizeGDB (http://www.maizegdb.org) and synthesized by AuGCT (Beijing, China). PCR amplification and product analysis were performed as reported previously [29].

To establish a genotype database of each individual, band patterns A, B, H, and U of the 100 SSR markers distributed on ten maize chromosomes were adopted. Pattern A indicated the origin of recurrent parent Zhao OP-6, B for non-recurrent parent CA339, H for heterozygous genotype, and U for unidentified genotype. According to the statistical analysis, the genetic background recovery rate of BC_6_F_3_ individuals was calculated with the formula G (*g*) = [L + X (*g*)] / (2L), in which G indicates the number of backcross generations, X indicates the number of molecular markers with the same pattern as parent Zhao OP-6, and L is the number of polymorphic SSR markers. The formula E [G(*g*)] = 1 - (1/2) ^*g*+1^ was used to calculate the theoretical genetic background recovery rate, and G referred to the number of backcross generations.

### Abundance analysis of *o2* and *wx* transcripts

Developing maize endosperm was collected from the center of three well-filled ears at 18 DAP. Total RNA was individually extracted with TRIZol kit (Thermo Fisher) and pooled together at the same amount. After DNA removal with RNase-free DNase I (Sigma-Aldrich), the cDNA was synthesized using an oligo d(T) primer kit (Promega). Two pairs of specific primers that spanned the exons of *o2* and *wx1* (O2RT-F: 5′-TCAGGAATAATCCAGTGCAGAA-3′ and O2RT-R: 5′-TCGACGTTAGCGTCGTTGTA-3′, and WXRT-F: 5′-TGTAGCTGCTTGCTTGTGCT-3′ and WXRT-R: 5′-CACCGAACAGCAGGGATTAT-3′) and one primer pair specific for the glyceraldehyde-3-posphate dehydrogenase (GAPDH) gene as the reference (GAPDH-F: 5′-CCCTTCATCACCACGGACTAC-3′ and GAPDH-R: 5′-AACCTTCTTGGCACCACCCT-3′) were designed for the analysis of transcript abundance. The PCR amplification products were separated on 1.5% agarose gels for analysis.

### Kernel characteristics and structure observation

The characteristics and appearance of intact kernels were collected by a SONY α700 camera (mode A). Endosperm hardness was graded from 1 to 5 following the CIMMYT universal standards, i.e., grade 1 (completely vitreous) to grade 5 (completely opaque), with a 25% difference between grades [30]. The kernel density was calculated by dividing the weight of 50 kernels by the volume (displacement of 95% ethanol in a cylinder) [31]. Mature kernels were peeled with a blade at the peripheral region, spray-coated with platinum, and observed under a Hitachi scanning electron microscope (SEM, S3400N). Developing kernels at 21 DAP were prepared as below: kernels (including partial pericarp) (1 mm × 3 mm × 1 mm) were sequentially fixed in 2.5% (w/v) glutaraldehyde and paraformaldehyde, followed by post-fixation in osmium tetraoxide. After dehydration in a gradient of ethanol (75−100%), samples were transferred to a propylene oxide solution and gradually embedded in paraffin. Sections of samples were prepared by a diamond knife microtome and observed under a Hitachi H7600 transmission electron microscope (TEM). Submicroscopic structure analysis was performed at the Institute of Food Science and Technology of Chinese Academy of Agricultural Sciences.

### Biochemical characterization and protein quantification

Mature kernels were dried at 65°C to constant weight and pulverized to a fine powder for the analysis of lysine and crude protein contents (at least two replicates and 40 g powder per sample). Crude protein content was measured with the Kjeldahl method according to the Chinese National Standard GB2905-82 (Nitrogen-to-protein conversion factor, K = 6.25). To determine the contents of 17 free amino acids (FAAs), all samples were pretreated following the Chinese National Standard GB7649-87, and analyzed by the S433D full-automatic amino acid analyzer (Beckman Coulter). The content of kernel crude oil was determined by using the Bruker Minispec MQ20 NMR Analyzer. Total starch was extracted, the content of amylose was measured by using the AUTOPOL III Polarimeter (Rudolph), and the percentage of amylopectin was calculated.

Zein and non-zein proteins were extracted according to the previous study with some modifications [32]. For each sample, a total of 15 mature kernels were collected from the centre of three well-filled ears (5 kernels from each ear), mixed, and soaked in distilled water for 6 h. Pericarps were then removed without damaging endosperm, and endosperm was further ground into a fine powder in liquid nitrogen. Powdered endosperm (50 mg per sample) was transferred to a 2 mL Eppendorf tube and incubated in 0.4 mL of extraction buffer (70% ethanol, 2% 2-mercaptoethanol, and 1% SDS) at 37°C with agitation for 2~3 h. After 10-min centrifugation (12,000 rpm) at room temperature (Centrifuge 5424R, Eppendorf), the supernatant comprised the zein fraction, and the sediment consisted of nonzein proteins. Extracted protein were measured using a bicinchoninic acid protein assay kit (Solarbio) according to the instructions. In order to identify the change of zein composition, aliquots of each supernatant (100 μL) were transferred to a fresh tube and dried at 50°C until the liquid evaporated absolutely. Distilled water (100 μL) was then added to dissolve the pellets. The same amounts of samples based on protein concentration were loaded onto a polyacrylamide gel (4% stacking gel and 15% separation gel), followed by staining with Coomassie Brilliant Blue R250 (Bio-Rad). Each sample had two replicates.

### 2D SDS-PAGE

Developing kernels at 18 DAP were used for protein extraction and 2-D SDS-PAGE analysis. The pooled endosperm powder (50 mg) was subjected to protein extraction using the acetone precipitation method [21] and dried in a lyophilizer at -20°C. The freeze-dried samples were then resuspended in IPG lysate buffer (0.2 M urea, 5% CHAPS, 50 mM thiocarbamide, 0.7% DTT, and 40% Bio-lyte) and incubated at 4°C for 1 h with frequent shaking. After removal of the insoluble fraction (12,000 rpm, 4°C and 15 min), soluble proteins were quantified by using a 2-D Quant Kit (GE Healthcare) with bovine serum albumin as a standard. IPG rehydration buffer (0.2 M urea, 5% CHAPS, 0.05 M thiocarbamide, 0.7% DTT, 40% Bio-lyte, and 1% bromophenol blue) was then added to adjust the final volume to 350~400 μL. The first dimension separation was performed using the Ettan IPG-phor-II (GE Healthcare). Aliquots of protein extract (~600 μg) were separated on 18 cm Immobiline Dry Strips (pH 5–8, Bio-Rad), with three technical replicates per sample. After rehydration for 14 h at 50 V, isoelectric focusing (IEF) was performed at 18°C following the procedures as shown below: 50 mA per strip and 250 V STEP for 1 h, 500 V STEP for 1 h, 2000 V GRAD for 30 min, 2000 V STEP for 1 h, 5000 V GRAD for 30 min, 5000 V STEP for 1 h, 8000 V GRAD for 2 h, 30000 V for focus and finally 500 V STEP for 10 h. The strips were then immersed in 8 mL of two types of equilibration buffer for 15 min with agitation. The immobilized proteins were subjected to second dimension separation on 12% SDS-PAGE gel at 16°C using the Mini-Protein II vertical gel apparatus (Bio-Rad). The conditions were: 1 W for approximately 30 min until the blue line reached the separation gel, and 10 W until the electrophoresis was finished. Proteins were visualized with Coomassie brilliant blue R250. Each sample had three technical replicates. Gels were compared, and spot intensities were quantified using the ImageMaster 2D Platinum 5.0 analysis software (Bio-Rad). Student’s *t*-test was used to determine the spot intensity changes between samples after normalization, and those with more than 1.4-fold changes were defined as significant difference (*p* < 0.05).

### Identification of protein spots by mass spectrometry

Protein spots that consistently appeared in three replicates and showed significant difference between samples were excised manually for mass spectrometry (MS) analysis. Briefly, gel pieces were destained in 25 mM NH_4_HCO_3_ and 50% (v/v) acetonitrile for 3 to 15 min at room temperature, freeze-dried, and rehydrated in 15 ng/μL sequencing-grade trypsin (Promega) at 4°C for 1 h. NH_4_HCO_3_ (25 mM) was then added to completely cover the gel pieces. After 16 h incubation at 37°C, the gel pieces were transferred to fresh Eppendorf tubes containing 5% trifluoroacetic acid and incubated at 37°C for another 1 h. Acetonitrile (50%) and trifluoroacetic acid (2.5%) were then added, and the mixtures were incubated at 37°C for 1 h. The freeze-dried peptides were analyzed by a Matrix Assisted Laser Desorption Ionization Time of Flight Mass Spectrometry (ABI-4800, AB Scienx) with the positive ion reflector mode and the sweep range of 900 to 4000 Da. Peaks derived from the mass spectra with a value of S/N > 10 were searched against the NCBI database by using the Mascot search engine. Only the protein spots with a score of over 70 (*p* < 0.05) were considered to be a putative protein and identified at Lab Assistant Biotechnology Company (Beijing, China).

## Results

### Polymorphism of SSR markers at target loci and MAS of *o2-wx* NILs

**Fig 1:**
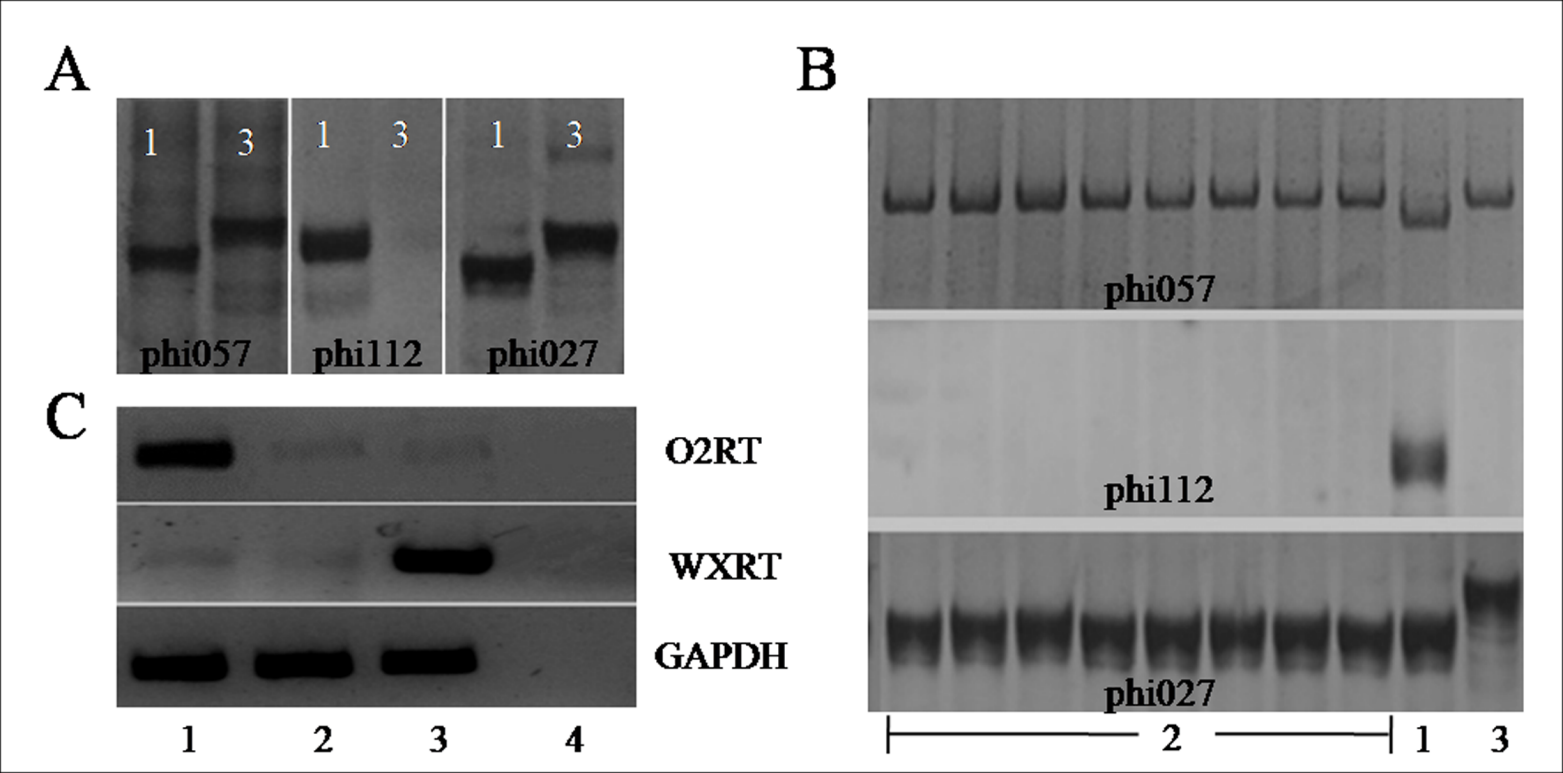
Electrophoresis analysis of SSR markers for MAS and transcript abundance of target genes. **(A)** SSR markers phi057, phi112 and phi027 specific for *o2* and transposable elements *rbg* and *wx1*, respectively. **(B)** The genotyping of different individuals from BC_6_ F_3_ family. **(C)** Transcript abundance analysis of *o2* and *wx1* in developing endosperm of 18 DAP by RT-PCR. O2RT and WXRT are two specific primers spanning the exons of *o2* and *wx1* allele, and GAPDH is the reference gene. 1, Zhao OP-6/*O2O2*; 2, *o2-wx* NIL individuals; 3, CA339; 4, blank control of distilled H_2_O.

Three polymorphic markers were tested in this study. As shown in Fig 1A, two markers (phi057 and phi112) specific for the *o2* allele were found to be polymorphic between the waxy maize Zhao OP-6/*O2O2* and the QPM CA339/*o2o2*, and phi027 of the *wx1* allele had polymorphism between the parents. Therefore, these polymorphic markers can be used for the MAS of corresponding target alleles: co-dominant markers phi057 and phi027 for the selection of heterozygous genotype *O2WX/o2wx* and recessive homozygous genotype *o2wx/o2wx*, while phi112 for the genotypes *O2O2* and *O2o2*. Genotype *O2WX/o2wx* was selected from the progeny and backcrossed to the recurrent parent Zhao OP-6/*O2O2*. After six backcrosses, recurrent parents and corresponding heterozygous plants had similar agronomic traits including stature, leaf, seed and flower. Finally, the recessive *o2wx*/*o2wx* plants were selected following two to three generations of self-pollination of *O2WX/o2wx* plants. The electrophoretic patterns of eight *o2-wx* NILs are shown in Fig 1B. The band pattern of Zhao OP-6/*o2o2* was consistent with that of CA339/*o2o2* and Zhao OP-6/*O2O2*, indicating that the *o2* allele has been successfully introgressed into the genetic background of Zhao OP-6/*O2O2*. Transcript abundance analysis (Fig 1C) indicated that the expression levels of *wx1* in Zhao OP-6/*o2o2* and Zhao OP-6/*O2O2* were identically low, while *O2* showed different expression levels. The transcript abundance of *o2* in Zhao OP-6/*o2o2* was similar to that in CA339.

To calculate the recovery rate of original genetic background of Zhao OP-6/*o2o2* individuals, 100 SSR markers were selected for polymorphism analysis. Of them, 54 were found to be polymorphic between parent plants. In the BC_6_F_3_ generation, the average recovery rate of selected individuals was 91.5%, 7.7% lower than the theoretical value (Table 1).

**Table 1:** Background analysis of two selected BC_6_F_3_ families.

BC_6_F_3_ family number	Recovery rate (%)	Donor parent genome (%)	Heterozygote genome (%)	Unidentified genome (%)
**Zhao OP-6/*o2o2*-1**	91.5	4.8	3.2	0.5
**Zhao OP-6/*o2o2*-2**	91.5	5.6	2.4	0.5

### Kernel characteristics and submicroscopic structure

**Fig 2:**
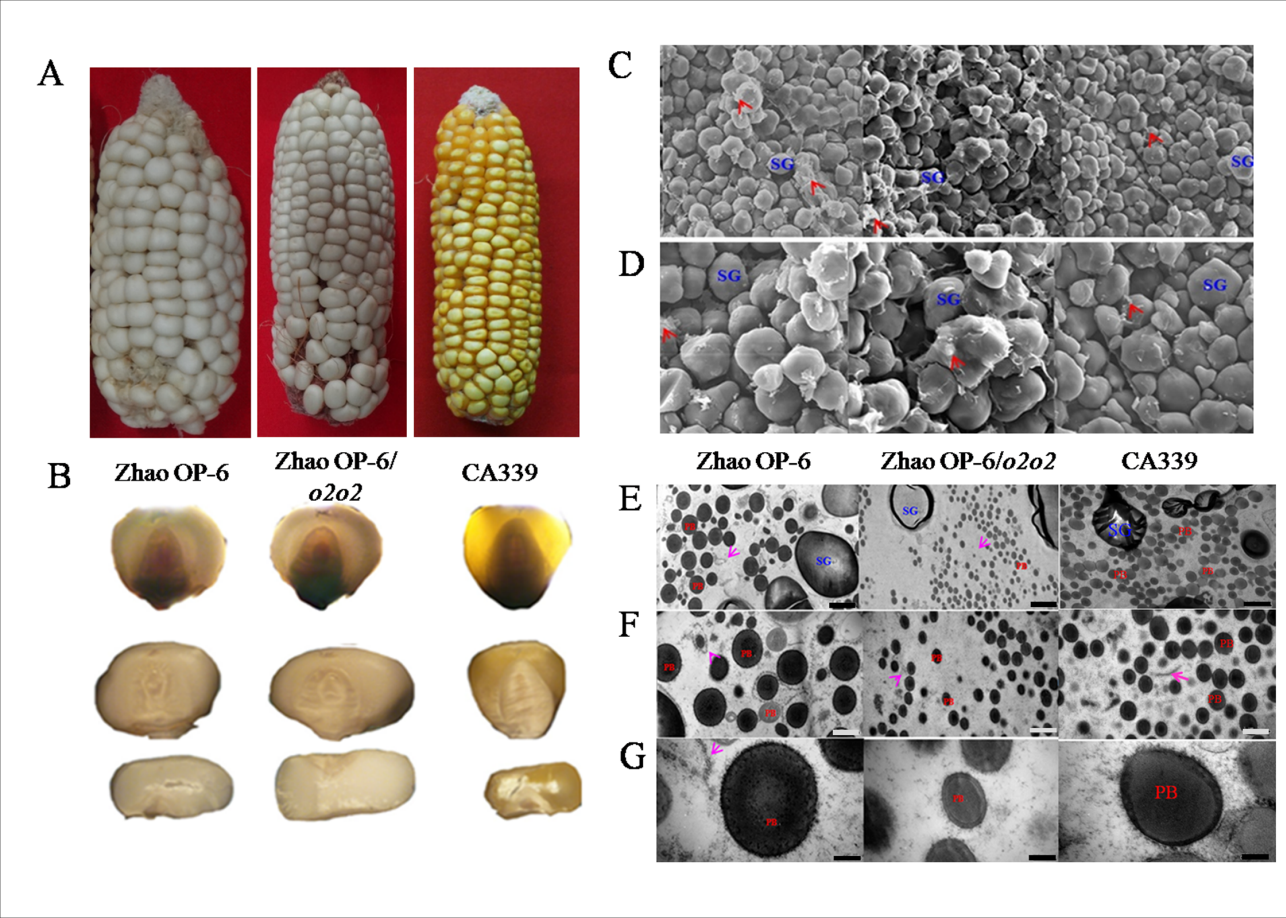
Phenotypic features of *o2-wx* NILs and the two parents. **(A)** Photographs of intact ears taken under normal light. **(B)** Light transmission of mature kernels on a light box. Kernels were randomly selected from the intact ears. **(C and D)** SEM of the peripheral region of mature endosperm at 1000 × and 2000 × magnification, respectively. SG, starch granule; red arrows, protein body. **(E**, **F and G)** TEM of the developing endosperms of 21 DAP at low (bars = 2 μm), moderate (bars = 1 μm) and high (bars = 200 nm) magnifications, respectively. PB, protein body; SG, starch granule; purple arrows, dilated ER in Zhao OP-6/*O2O2*.

Under normal and transmitted light, the kernels of Zhao OP-6/*o2o2* and Zhao OP-6/*O2O2* were completely vitreous indicated that the hardness of the two lines were 1. (Fig 2A and 2B). In addition, no significant difference was detected in the hundred-kernel weight and kernels density (S1 Table). Under SEM, the starch granules of Zhao OP-6/*o2o2* were compact and embedded in a dense proteinaceous matrix (Fig 2C), while those of Zhao OP-6/*O2O2* and CA339 had relative smooth, loosely packed starch granules (Fig 2C) with little contact with protein bodies (Fig 2D). The dense packing of protein bodies around starch grains may account for the more vitreous endosperm [33]. Immature endosperm cells of Zhao OP-6/*o2o2* and Zhao OP-6/*O2O2* at 21 DAP showed similar micro- and ultra-structures (Fig 2E and 2F), in which protein bodies were regularly shaped, well separated from each other, and evenly surrounded by starch granules. Notably, smaller protein bodies accumulated in Zhao OP-6/*o2o2* endosperm cells at the volume of one third of that of the parents, but at higher amount (Fig 2G). Moreover, numerous small and cisternal ERs were dilated in the endosperm cells of recurrent parent Zhao OP-6/*O2O2* and accumulated on the peripheral cell walls with polygonal ring or vesicle-like structures (Fig 2F). These structures may originate from the endo-membrane system.

### Changes of zeins and FAA composition in *o2-wx* NILs endosperm

**Fig 3:**
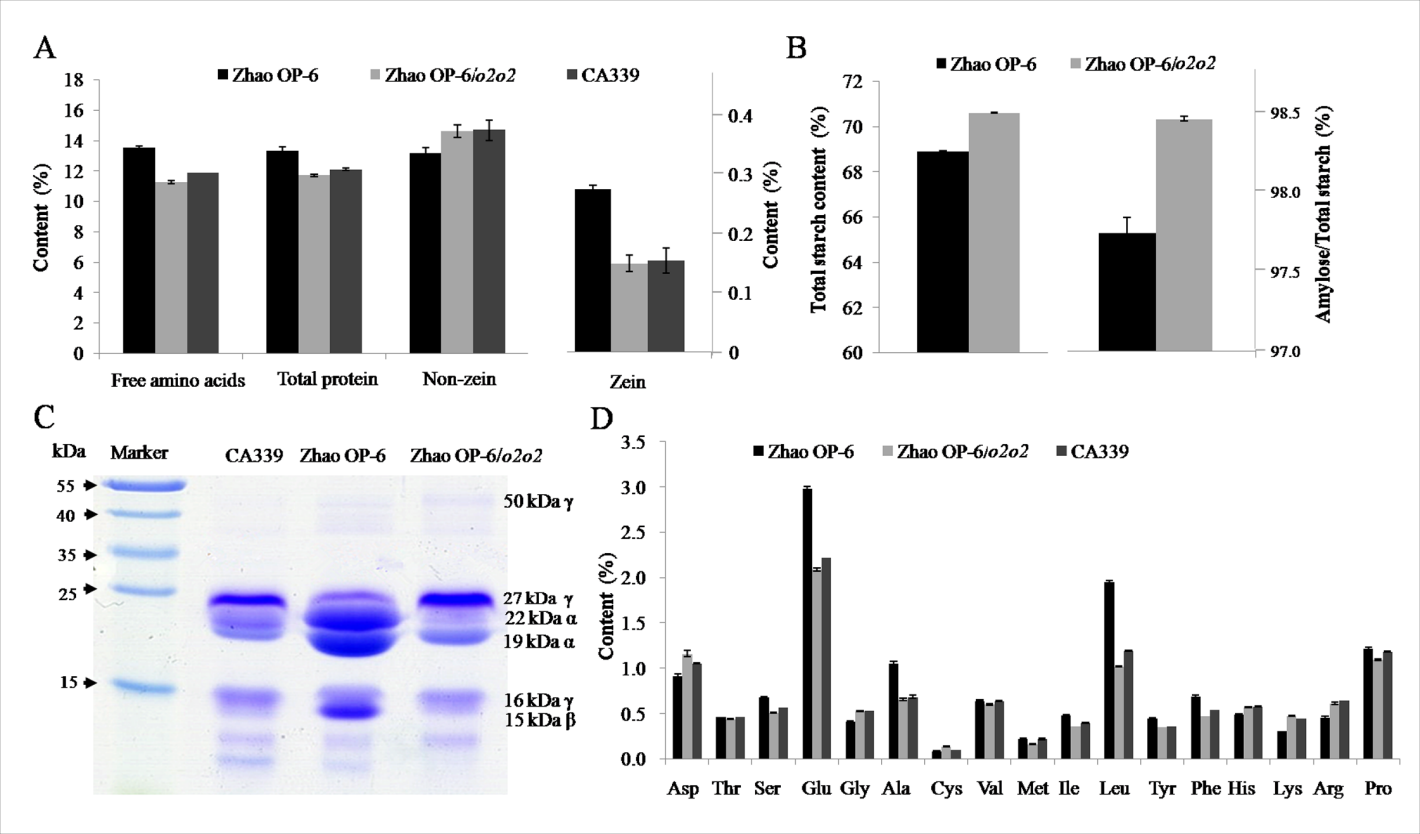
Biochemical characterization of *o2-wx* NILs and the two parents. **(A)** The contents of FAAs, total protein, zein and non-zein and in mature kernels. The data were calculated based on the percentage per milligram of dried mature kernel. **(B)** Contents of total starch and amylopectin in the mature kernels of Zhao OP-6/*O2O2* and Zhao OP-6/*o2o2*. The data were calculated based on per milligram of dried mature kernels. **(C)** SDS-PAGE analysis of zein proteins extracted from mature endosperm. **(D)** The contents of 17 FAAs in mature kernels. All data are shown as mean ± S. E. (*n* = 3).

To investigate the potential biochemical differences between Zhao OP-6/*o2o2* and the parents, we studied the major characters and components of mature kernels. No significant difference was found in the contents of moisture and oil between Zhao OP-6/*O2O2* and Zhao OP-6/*o2o2* (S1 Table), although these lines showed difference from CA339 in moisture and oil contents and hardness. However, in comparison to parent plants, Zhao OP-6/*o2o2* showed reduced FAAs but increased total starch contents (Fig 3A and 3B). Moreover, the percentage of amylopectin of Zhao OP-6/*o2o2* was 98.5%, higher than that of Zhao OP-6/*O2O2* (97.7%, *p* < 0.01, Fig 3B).

The qualitative and quantitative differences of zein, non-zein proteins and FAAs of different maize lines were also assessed and compared. A shown in Fig 3A, there is no significant difference in the total protein contents of Zhao OP-6/*o2o2* and Zhao OP-6/*O2O2*. However, decreased amounts of zeins and increased amounts of non-zeins were found in Zhao OP-6/*o2o2*. SDS-PAGE (Fig 3C) indicated that Zhao OP-6/*O2O2* had five major polypeptides in endosperm, i.e. 27-kDa γ-zein, 22-kDa α-zein, 19-kDa α-zein, 16-kDa γ-zein, and 15-kDa β-zein. Four of them (except for the 27-kDa γ-zein) were also found in Zhao OP-6/*o2o2* but with decreased amounts, especially the 22-kDa α-zein and 15-kDa β-zein. The reduced accumulation of 19-kDa α-zein had also been reported in a previous study [16]. These two α-zeins showed considerably reduced accumulation in the donor parent CA339. Notably, in comparison with Zhao OP-6/*O2O2*, the content of 27-kDa γ-zein was significantly increased in Zhao OP-6/*o2o2*, which is similar to CA339 [34]. The unchanged level of 16-kDa γ-zein ruled out the possibility that expression of zein gene was generally affected in Zhao OP-6/*o2o2*. Comparison of kernel FAA composition also revealed differences between Zhao OP-6/*o2o2* and the parents (Fig 3D). In total, Zhao OP-6/*o2o2* had 16.7% less FAAs than recurrent parent Zhao OP-6/*O2O2*. In the mature kernels of Zhao OP-6/*o2o2*, the contents of lysine and glycine were most significantly increased by 51.6% and 26.9%, respectively, while threonine, cysteine, and methionine derived from the aspartic acid pathway had no change in amounts. Though the two glutamic acid-derived amino acids, histidine and arginine, showed no difference between Zhao OP-6/*o2o2* and Zhao OP-6/*O2O2*, the content of glutamic acid decreased in Zhao OP-6/*o2o2*. Besides, the contents of leucine, serine and alanine were significantly decreased by 47.8%, 24.9% and 38.1%, respectively. And Zhao OP-6/*o2o2* had slightly decreased contents of isoleucine, tyrosine, and phenylalanine.

### Proteomic comparison of parent plants and *o2-wx* NILs

**Fig 4:**
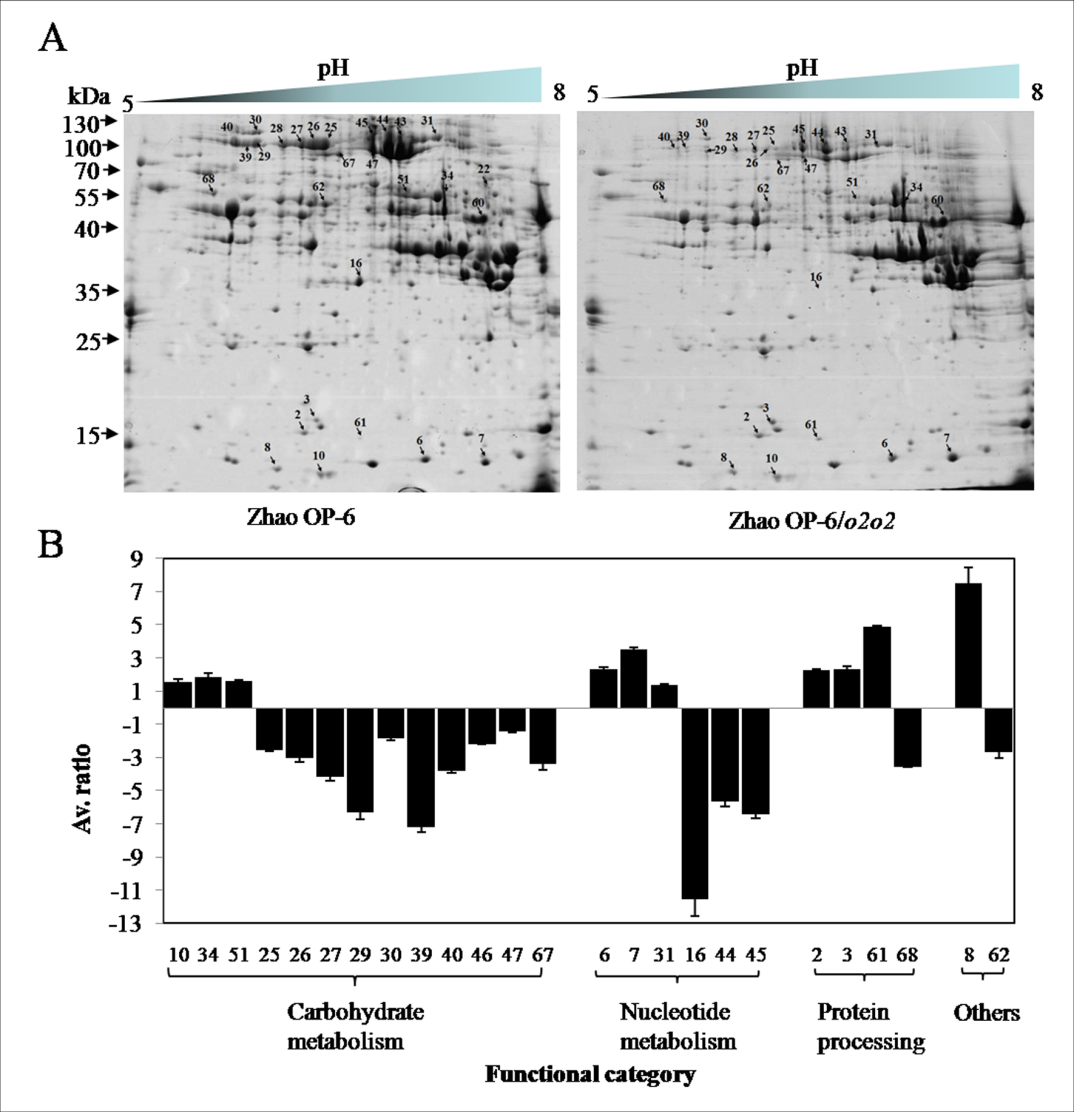
2-D SDS-PAGE analysis of the polypeptides in developing endosperms of *o2-wx* NILs and parent plants at 18 DAP. **(A)** Profiles of Zhao OP-6/*O2O2* (left) and Zhao OP-6/*o2o2* (right). Protein spots showing significant differences (1.4-fold, *p* < 0.05) were numbered and identified by MS. **(B)** Quantitative analysis of the proteins of differential abundance. Values are shown as mean ± S.E. (*n* = 3).

2-D SDS-PAGE was used to compare the proteins in maize endosperms. As results, the molecular weights of maize endosperm proteins ranged from 10 to 130 kDa with the pH gradient from 5 to 8 (Fig 4A). A total of 40 protein spots displayed significant abundance differences or showed altered accumulations at the protein level (Fig 4B). Except for unidentified protein spots (due to weak spectra or unsuccessful database searches), 25 protein spots were identified by MS (S2 Table). A comparison of protein abundance between Zhao OP-6/*o2o2* and parent plants showed that fewer protein species (10 of 25 protein spots) were up-regulated in Zhao OP-6/*o2o2*. Of them, proteins involved in the maintenance and folding of proteins in the ER and defense to biotic and abiotic stresses were enriched, including the 17.5 kDa class II heat shock protein (spot 2), 17.4 kDa class I heat shock protein (spot 3), nucleoside diphosphate kinase 1 (NDPK1, spots 6 and 7) and 17.0 kDa class II heat shock protein (spot 61). Moreover, the 14 kDa zinc-binding protein (ZBP14, spot 8), trypsin/factor XIIA inhibitor (TPA, spot 10) and glucose-1-phosphate adenylyltransferase (ADPase, spots 34 and 51) showed increased abundances in Zhao OP-6/*o2o2*, which were probably related to the improved zinc site binding and starch synthesis. On the other hand, the down-regulated proteins in Zhao OP-6/*o2o2* might be involved in different metabolic pathways. The cyPPDK1-related proteins (spots 25, 27, 29, 39, and 40) were predominant with similar fold changes and accounted for 24% of the differential proteins. The significant reduction in the accumulation of sucrose synthase 1 (SH1, spots 46 and 47), a key enzyme in glycometabolism, indicated the changes of sugar metabolism in Zhao OP-6/*o2o2*. The α-glucan phosphorylase 1 (PHS1, spot 30) and 1,4-α-glucan-branching IIb (SBE IIb, spot 67) that are involved in starch metabolism were both down-regulated in Zhao OP-6/*o2o2*. Although several proteins involved in protein folding and plant defense were enriched in Zhao OP-6/*o2o2*, the expression of Chaperonin 60 (CPN60, spot 68) and RIP (spot 16) was completely suppressed. The suppression of RIP might be associated with the low expression levels of EF2-related proteins (spots 31, 44, and 45). S-adenosyl-L-homocysteine hydrolase (SAHH, spot 62) that takes part in methylation by regulating methyl transferase reactions was also down-regulated in Zhao OP-6/*o2o2*. The differential expression of these proteins in Zhao OP-6/*o2o2* and parent plants may explain the principle biochemical and morphological variations of *o2-wx* NILs.

## Discussion

In the present study, MAS technique was used to produce a set of *o2-wx* NILs by introgressing *o2* allele into waxy maize line Zhao OP-6/*O2O2*. Recurrent parents and the *o2-wx* NILs (Zhao OP-6/*o2o2*) had similar agronomic traits including stature, leaf, seed and flower. However, some specific fragments of CA339 were also found in Zhao OP-6/*o2o2* by calculating the recovery rate of original genetic background. The result indicated that it is impossible to produce NILs harboring single recessive allele *o2-wx* only based on recurrent phenotypes and MAS using a single marker. Therefore, a larger number of markers are indispensable to create single allele NILs by MAS for foreground and background selections [27].

Endosperm of *o2* mutants is usually soft, fragile, rich in water, and susceptible to pests [19]. However, some endosperm modifier alleles having capacity of changing opaque endosperm into vitreous one can mitigate these weaknesses [35]. In the present study, Zhao OP-6/*o2o2* was found to share similar kernel phenotype to the recurrent parent, such as vitreousness, hardness, density and hundred-kernel weight, suggesting that the endosperm modifier *o2m* allele may play a crucial role in the waxy genetic background (S1 Table). Moreover, the *wx1* allele has been identified to be positively correlated with the vitreousness of maize endosperm and thus may represent another endosperm modifier allele [36]. SEM analysis indicated that starch grains, protein bodies, and viscous cytoplasm in combination formed a more compact matrix in the vitreous region of Zhao OP-6/*o2o2* than the parents. Normally, maize starch granules do not form cohesive contacts with one another. However, there are physical connections between starch granules of Zhao OP-6/*o2o2*, which may account for the hard, vitreous phenotype. Meanwhile, accumulation of protein bodies contributes to a more vitreous and harder endosperm of maize [37]. TEM further revealed the configuration and distribution of protein bodies. In Zhao OP-6/*o2o2* endosperms at 21 DAP, smaller and regularly shaped protein bodies were found to prominently aggregate into clumps. Interestingly, though Zhao OP-6/*o2o2* endosperm cells accumulated smaller protein bodies at one third of the parent volume, the number of the protein bodies was higher than that of the parents. This observation corresponds to the putative role of increased 27-kDa γ-zein in CA339 endosperm [34]. The 27-kDa γ-zein is located on the periphery of protein bodies and is widely cross-linked by disulfide bonds and covalent linkages between protein bodies; these interactions provide a mechanism for cementing protein bodies around starch grains [38]. Moreover, the 27-kDa γ-zein has been demonstrated to play a key role in endosperm modification by RNAi and γ-radiation; its accumulation contributes to a rigid matrix vitreous endosperm as well [34,39]. Notably, dilated rough ER was observed surrounding the protein bodies in Zhao OP-6 endosperm cells, indicating that ER stress is probably induced in plant cells by a variety of factors, including agents affecting calcium homeostasis, abiotic and biotic stresses, and inhibitors of glycosylation [40,41]. ER stress usually occurs in *o2* mutants, and leads to a non-homeostasis environment for protein folding and formation of disulfide bonds in ER lumen [42,43]. However, ER stress was rarely found in Zhao OP-6/*o2o2*. The reason might be that several proteins involved in the maintenance and folding of proteins in the ER are up-regulated due to the alteration of protein body structure in ER.

The major pathways regulated by O2 are amino acid biosynthesis and starch-protein balance. Typically, FAAs constitute about 1 to 3% of the non-protein nitrogen in wild-type maize endosperm whereas exhibit increased levels in *o2* mutants [44–46]. In the present study, the contents of FAAs in Zhao OP-6/*o2o2*, especially leucine, serine and alanine derived from the glycolytic intermediates and most abundant in the early endosperm development, appeared to have a genetic predisposition for down-accumulation. This result is against the previous study [45], indicating that the *wx1* as an *opaque2* modifier may influence the amino acid composition in maize endosperm [36]. Reduction of these abundant amino acids, especially leucine, is largely responsible for the relative increase of minor amino acids, especially lysine and glycine, as the endosperm matures. The increased level of lysine in Zhao OP-6/*o2o2* largely depended on the reduction of α-zein synthesis which excludes lysine while several non-zein proteins which accounts for most of the higher percentage of lysine are associated with varying degrees of increased accumulation [47]. In addition, O2 has the ability to regulate the activity of cyPPDK1 and AHAS [48]. AHAS catalyzes the first step of branched amino acid synthesis, and cyPPDK1 is a key regulator of the glycolytic pathway derived from pyruvate; both enzymes were also down-regulated by *o2* and finally accounted for the reduction of leucine concentration [21,49].

Pyramiding of recessive alleles, especially double-recessive or triple-recessive mutations, has specific genetic effects on ameliorating the quantity and quality of starch, sugar, oil, and protein in endosperm [50]. A previous study reported that the double-recessive *o2* and *wx1* mutations had no effect on the grain starch content [26]. However, in the present study, the total starch content of Zhao OP-6/*o2o2* kernels was significantly higher than the recurrent parent Zhao OP-6. Interestingly, the amylopectin content of Zhao OP-6/*o2o2* was also very significantly higher than that in Zhao OP-6/*O2O2*. These results demonstrated that the altered starch structure by completely suppressing the expression level of *wx1* in *o2-wx* NILs may elucidate the role of GBSS I in production of amylopectin. Compared with Zhao OP-6/*O2O2* and soft *o2* genotypes, amylopectin in Zhao OP-6/*o2o2* with reduced intermediate-length α-1,4-linked glucose chains is associated with increased swelling in water and formation of tight contacts between starch granules [33]. In addition, several starch biosynthesis genes, especially ADPase and SBE IIb, showed altered expression levels between Zhao OP-6/*o2o2* and Zhao OP-6/*O2O2* based on 2D SDS-PAGE. ADPase [51] that catalyzes the first committed step of starch synthesis was up-regulated, while SBE IIb [52] that catalyzes the formation of α-1,6-linked glucan and is required for amylopectin synthesis at the surface of the starch granule was down-regulated. Previous studies indicated that the deficiency of SBE Ia has no impact on endosperm starch structure, whereas SBE IIb is closely related to the reduced level of amylopectin [53]. But there is no evidence to verify the predominant function of SBE IIb over SBE Ia, and SBE Ia might have effect on amylopectin structure only in the absence of SBE IIb [54]. In *vitro* assay, SBE Ia preferentially produces longer polymers (> 16) while SBE IIb produced shorter ones (< 12) [55]. Thus the increased amylopectin level may largely depend on the SBE Ia activity. In addition, expression changes of one or more starch biosynthesis enzymes probably results in the starch modification of Zhao OP-6/*o2o2*, or altering the expression or mutation of one starch biosynthetic enzyme has multiple effects on enzyme activities.

As highlighted before, the development of maize endosperm is complex, which is driven by qualitative and quantitative coordinate expression of endosperm protein asset of different genotypes [21]. To better clarify the role of O2 in endosperm protein expression and to investigate their possible interactions in waxy genetic background, 2D SDS-PAGE analysis of 18 DAP endosperms was performed. Of 25 differentially expressed protein spots, those putative cyPPDK1 spots represents an exception that showed similar down-regulation (up to 3-fold or higher). Although cyPPDK1 isoforms had slight changes in p*I*s and molecular weights, they were all controlled by O2 as shown before [56]. A close examination of the expression patterns of proteins involved in sugar and starch metabolism shows that Zhao OP-6/*o2o2* has perturbations in sucrose metabolism and environmental response. Sucrose has been shown to act as signals to trigger changes in the expression of a broad range of post-transcriptional modifications [57]. Therefore, the differential expression of SH1, a key enzyme in sucrose synthesis pathway, indicated that genetic backgrounds of different lines or different environment responses may indirectly affect the expression of SH1 in *o2* mutants. Moreover, the expression levels of several proteins involved in maintenance and folding of proteins in ER showed cross-occurrence between Zhao OP-6/*o2o2* and Zhao OP-6/*O2O2*. For example, the small cytoplasmic chaperones (heat shock protein) were significantly up-regulated in Zhao OP-6/*o2o2*, whereas the expression of Chaperonin 60 was down regulated. These proteins are related to the protein unfolding, and their interaction is likely related to protein body distribution and structure in the ER [42]. Stress response and defense-related proteins including NDPK1 and RIP also showed differential expression in Zhao OP-6/*o2o2*. NDPK1 is a master regulator of diverse pathways in the cell and plays a important role in plant defense responses [58], while RIP has a defensive role against pathogens and viruses and represents a well-known target of O2 regulation [59]. In Zhao OP-6/*o2o2*, the expression level of NDPK1 was increased by about 2-fold, while RIP was completely suppressed. The up-regulation of NDPK1 is more likely related to pleiotropic responses instead of resistance to pests since evidences have shown that *o2* mutants are much more sensitive to pests [19]. The decrease in RIP abundance may be associated with the low expression of EF2; as a result, the efficiency of translation was suffocated. Regulation of protein expression is mainly controlled by signal transducers, which is central to a myriad of biological processes at the molecular level [24]. In the present study, two proteins, SAHH and ZBP14, were identified to involve in the signal transduction. SAHH is an NAD^+^-dependent tetrameric enzyme which catalyzes the breakdown of *S*-adenosyl homocysteine to homocysteine and adenosine. The decreased accumulation of SAHH in Zhao OP-6/*o2o2* may affect cell growth and regulation of gene expression [60]. ZBP14 as one of the key enzymes in cellular signal transduction has a central role in the control of many cellular processes, and its activity is modified by activators such as diacylglycerol [61]. Although further studies are required to provide convictive proofs that O2 regulates the differential expression of proteins directly or indirectly, the interaction of these proteins is a strong indicator to facilitate the construction of specific phenotype and influence the functions of endosperm cells.

## Supporting Information

S1 Table Kernel characters of maize lines in this study. (XLSX)

S2 Table Differential proteins (*p* < 0.05) in the developing endosperm (18 DAP) of maize lines Zhao OP-6/*O2O2* and Zhao OP-6/*o2o2*. (XLSX)
