# Correction: Introgression of *opaque2* into Waxy Maize Causes Extensive Biochemical and Proteomic Changes in Endosperm

**DOI:** 10.1371/journal.pone.0161924

**Published:** 2016-08-23

**Authors:** Zhiqiang Zhou, Liya Song, Xiaoxing Zhang, Xinhai Li, Na Yan, Renpei Xia, Hui Zhu, Jianfeng Weng, Zhuanfang Hao, Degui Zhang, Hongjun Yong, Mingshun Li, Shihuang Zhang

There is an error in the Funding Disclosure statement. The correct Funding Disclosure statement should read: "This work was supported by the National Natural Science Foundation of China (Grant No. 31401390) and the China Agriculture Research System (Grant No. CARS-02-01). The funders had no role in study design, data collection and analysis, decision to publish, or preparation of the manuscript."
